# Priorities for family building among patients and partners seeking treatment for infertility

**DOI:** 10.1186/s12978-017-0311-8

**Published:** 2017-04-05

**Authors:** Elizabeth A. Duthie, Alexandra Cooper, Joseph B. Davis, Jay Sandlow, Katherine D. Schoyer, Estil Strawn, Kathryn E. Flynn

**Affiliations:** 1 Center for Patient Care and Outcomes Research, 8701 Watertown Plank Rd, Milwaukee, WI 53226 USA; 2 Social Science Research Institute, Duke University, Box 90989, Durham, NC 27708 USA; 3 Reproductive Medicine Associates of New York, 635 Madison Ave, New York, NY 10022 USA; 4 Department of Urology, Medical College of Wisconsin, 9200 W Wisconsin Ave, Milwaukee, WI 53226 USA; 5 Department of Obstetrics & Gynecology, Division of Reproductive Endocrinology and Infertility, Medical College of Wisconsin, 9200 W Wisconsin Ave, Milwaukee, WI 53226 USA

**Keywords:** Infertility, Fertility, Couples, Patient-centered care, Decision making

## Abstract

**Background:**

Infertility treatment decisions require people to balance multiple priorities. Within couples, partners must also negotiate priorities with one another. In this study, we assessed the family-building priorities of couples prior to their first consultations with a reproductive specialist.

**Methods:**

Participants were couples who had upcoming first consultations with a reproductive specialist (*N* = 59 couples (59 women; 59 men)). Prior to the consultation, couples separately completed the Family-Building Priorities Tool, which tasked them with ranking from least to most important 10 factors associated with family building. We describe the highest (top three) and lowest (bottom three) priorities, the alignment of priorities within couples, and test for differences in prioritization between men and women within couples (Wilcoxon signed rank test).

**Results:**

Maintaining a close and satisfying relationship with one’s partner was ranked as a high priority by majorities of men and women, and in 25% of couples, both partners ranked this factor as their most important priority for family building. Majorities of men and women also ranked building a family in a way that does not make infertility obvious to others as a low priority, and in 27% of couples, both partners ranked this factor as the least important priority for family building. There were also differences within couples that involved either men or women ranking a particular goal more highly than their partners. More women ranked two factors higher than did their partners: 1) that I become a parent one way or another (*p* = 0.015) and 2) that I have a child in the next year or two (*p* < 0.001), whereas more men ranked 4 factors higher than their partners: 1) that our child has [woman’s] genes (*p* = 0.025), 2) that our child has [man’s] genes (*p* < 0.001), 3) that I maintain a close relationship with my partner (*p* = 0.034), and 4) that I avoid side effects from treatment (*p* < 0.001).

**Conclusions:**

Clinicians who support patients in assessing available family-building paths should be aware that: (1) patients balance multiple priorities as a part of, or beside, becoming a parent; and (2) patients and their partners may not be aligned in their prioritization of achieving parenthood. For infertility patients who are in relationships, clinicians should encourage the active participation of both partners as well as frank discussions about each partner’s priorities for building their family.

## Plain English summary

Many couples who are unable to conceive a baby seek medical advice from infertility specialists. Even with that guidance, couples face difficult choices as they try to build their families. While we know that they want to have a baby, researchers know very little about how couples balance other priorities that could influence their decisions about whether to pursue treatment and what treatment will best meet their goals.

To learn more about couples’ priorities, we created a list of 10 factors related to family-building decisions. We recruited 118 people (59 couples) who planned to see a reproductive specialist and asked them to each separately rank the importance of the 10 factors. Then we looked for similarities and differences in the priorities of men and women within couples.

We found that there were differences between men and women within couples for six of the 10 factors: becoming a parent one way or another; passing on the woman’s genes; passing on the man’s genes; having a child within a year or two; maintaining a close relationship; and avoiding treatment side effects. For two factors, partners in >25% of couples ranked the factors exactly the same: maintaining a close relationship (highest priority) and building a family in a way that doesn’t make infertility obvious to others (lowest priority).

We recommend that infertility specialists be aware that the couples they treat are balancing many priorities and that partners may not agree about how to balance those priorities and that they should counsel them accordingly.

## Background

While medical decision making is often difficult, several features of medical treatment for infertility make these decisions especially challenging. For example, because health insurers are not mandated to cover infertility treatment in 35 of the United States (US), cost is thought to be a major consideration for most Americans considering infertility care [1, 2]. Because of uncertainty about whether any particular treatment will ultimately lead to a live birth, the upfront cost raises the stakes of treatment decisions for couples – a decision to invest in one path may limit the resources available to pursue other options if a treatment is unsuccessful. Other factors must also be weighed, such as the importance of a genetic connection to a future child, experiencing pregnancy and childbirth, and the potential for treatment side effects for the parent or child. Because various family-building paths are associated with these factors in different ways, the relative value a hopeful parent places on any given priority may point toward some paths while excluding others.

Additionally, treatment-related decisions about infertility necessarily involve more than one actor. Even when a couple is in agreement about seeking care to start a family, partners may not agree about where to set limits in terms of financial outlay or time invested, how to prioritize genetic parentage, or what treatment-related risks are acceptable. The question of how couples reach joint decisions is one that has been studied extensively and from a variety of perspectives, including game-theoretic [3–5], social-psychological [6], and sociological [7–11]. Extensive applications exist focusing on topics from relocation decisions among two-earner couples [12], to consumer behavior [13], to contraceptive use [14], and sexual relations [15].

Yet despite the potential for patients’ relative valuation of family-building priorities to affect infertility treatment decisions, little research literature addresses this topic. Previous research has examined a related concept of couples’ motivations and goals for childbearing and parenting with attention to the impact of infertility. In a study of 214 couples, Miller et al. found that infertile couples considering the use of assisted reproductive technology (ART) were more highly motivated by perceived positive aspects of parenthood and less concerned with perceived negative aspects of parenthood compared to couples with no known fertility problems [16]. Langdridge et al. compared parenthood motivations among 10 pregnant couples with no known fertility issues, 10 couples with infertility who were pursuing in vitro fertilization, and 10 couples with infertility who were pursuing donor insemination [17]. The three groups were more similar than different in terms of their reasons for pursuing parenthood, with respondents overwhelmingly endorsing a core “triad” of reasons to pursue parenthood: giving love, receiving love, and added enjoyment/fun in life. A phenomenological analysis of three couples over six months after beginning treatment with in vitro fertilization found that couples balanced their main goal of achieving parenthood with four other goals: biological parenthood, retaining emotional well-being, remaining financially secure, and maintaining good relationships with partners [18]. Finally, Thompson et al. found that in 37 couples seeking infertility treatment, both partners reported placing similar levels of importance on reaching the goal of parenthood [19]. These previous studies focused on general motivations for becoming a parent; to our knowledge no existing studies have examined specific factors related to achieving parenthood for men and women who are currently experiencing infertility. This is important since couples who are experiencing infertility may have individual values and preferences but must make joint decisions in the context of finite time and resources for family building.

Our objectives in this study were to describe how men and women in the early stages of seeking medical treatment for infertility prioritize different factors related to infertility decision making and to test for differences in priorities between partners within couples. We also present a novel tool to help individuals consider their priorities; the tool may also be useful for facilitating discussions about priorities with partners and providers.

## Methods

A convenience sample of new patients at a Reproductive Medicine Center affiliated with a large academic medical center in suburban Milwaukee, Wisconsin was recruited between May of 2013 and June of 2014. Letters detailing the research study were mailed to 613 patients who had first-consultations scheduled at least one week in the future with a reproductive specialist (RS), specifically, either a reproductive endocrinologist and infertility specialist or a fellowship-trained reproductive urologist. Because of the short window of time to recruit to the study before the first appointment, people were only invited to participate once; no follow-up attempts were made. After receiving the letter, 155 patients contacted the study team to learn more about the study. We wanted to understand the experiences of couples who were naïve to specialty treatments for infertility, thus additional inclusion criteria included not having previously had a child using any ART, not having previously tried IVF, and the ability to provide data before the first appointment with the RS. One hundred eleven people met these criteria, and 92 patients and 68 of their partners enrolled in the study. For this analysis we included the 59 opposite-sex couples for whom we had data on the Family-Building Priorities Tool. All participants provided informed consent. The study was approved by the Medical College of Wisconsin/Froedtert Hospital Institutional Review Board.

### The Family-Building Priorities Tool

**Fig. 1:**
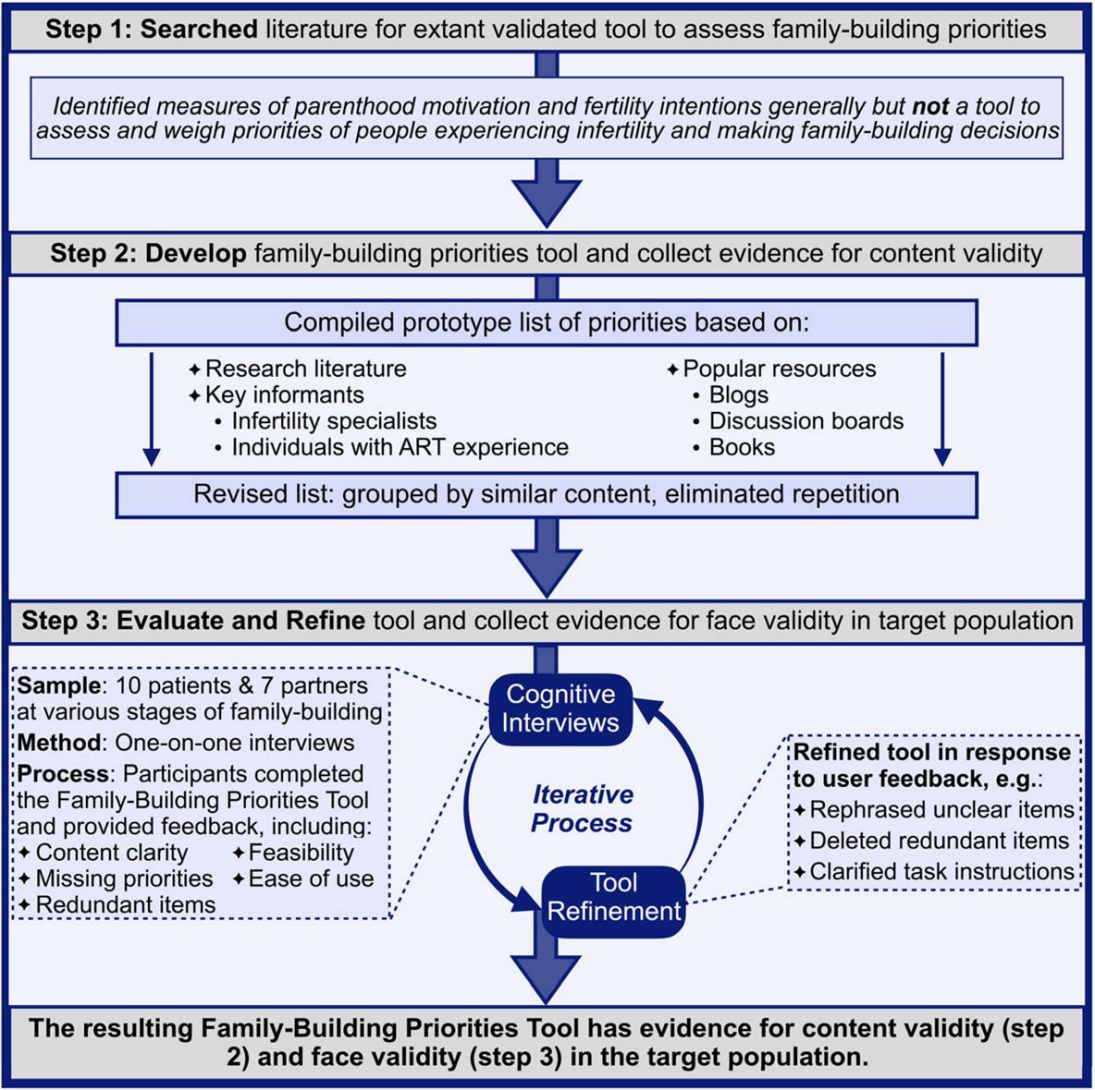
Development and validation of the Family-Building Priorities Tool

We were unable to find an extant tool to assess family-building priorities for people experiencing infertility. Thus we created the Family-Building Priorities Tool (Table 1). The development process is shown in Fig. 1. Available family-building options for couples experiencing infertility require trade-offs, so we wanted to assess the relative weight that individuals experiencing infertility place on different factors rather than asking them to rate how important each one is. The Tool instructs individuals to rank factors in order of importance from 1 to 10. Conceptually these priorities are not meant to represent a single construct or latent variable; as such, psychometric evaluation looking at internal consistency, reliability, or factor structure was not appropriate. We developed and evaluated the validity of the Tool as follows. First, we developed a list of candidate priorities after a review of the scientific literature [16, 17, 20, 21] and popular infertility resources [22] and in consultation with the physicians and patients experienced with ART who were part of the study team. This process resulted in a prototype Tool with evidence for content validity. We then evaluated the prototype Tool using cognitive interviews. Cognitive interviews apply techniques from cognitive theory to systematically evaluate and revise questionnaire items through intensive verbal probing [23]. We conducted a total of 17 interviews with ten women and seven men recruited from the same Reproductive Medicine Center described above, but we specifically targeted individuals at all stages of the process of infertility decision making. During the cognitive interviews we asked participants to complete the Tool. Then we examined the instructions and each priority in turn, asking participants to rephrase priorities in their own words to evaluate comprehension, to add any priorities to the list that they thought were missing, and to share any other thoughts or ideas that came to mind while examining the Tool. Cognitive interviews were conducted iteratively, that is, after revisions were made to the Tool we retested the revised version in additional interviews. These cognitive interviews provided evidence for face validity of the Tool.

**Table 1:**
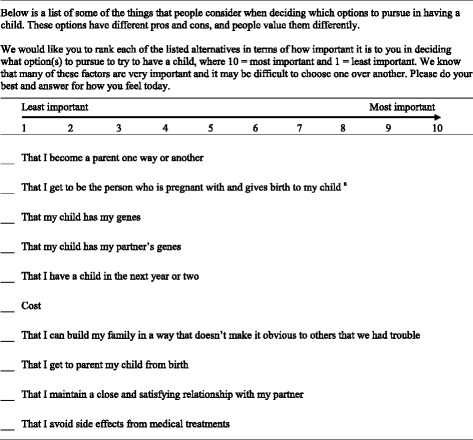
Family-Building Priorities Tool

^a^The wording for this item varied depending on the respondent’s role in the couple. Women were presented with this item as it is worded in the table above. The wording for men was slightly adjusted: “That my partner gets to be the person who is pregnant and gives birth to my child”

### Data collection and analysis

Each participant completed a self-administered questionnaire using REDCap [24] prior to the first scheduled consultation with the RS (median three days; interquartile range = one to six days).

We compared sociodemographic and self-reported health characteristics between women and men accounting for the non-independence of the samples using Wilcoxon’s signed-rank test (ordinal variables), McNemar’s and extended McNemar’s tests (categorical variables), and paired t-tests (continuous variables). In order to broadly summarize which factors were most and least important to participants, we describe the percentages of men and women who identified each of the 10 factors as a high (top three) or as a low (bottom three) priority, and within the groups of men and women, we also identify factors for which a sizeable proportion (we chose 25%) designated it as a high priority and at least as many designated it as a low priority. Given the non-independence of this data, we do not test for differences by women and men as groups. For each factor we show the percentage of couples who ranked it identically, and we used the Wilcoxon signed-rank test to assess whether male and female partners within couples ranked each factor differently from one another. We considered a two-tailed α level of 0.05 significant. Extended McNemar’s tests were performed using SAS version 9.4 (SAS Institute, Cary, NC). All other analyses were performed using Stata 14 (Stata Corp., College Station, TX).

## Results

### Sample characteristics

Over half of women in the sample were less than 35 years of age at the time of their first scheduled consultation, and most identified as white, non-Hispanic, had at least a bachelor’s degree, were employed full-time, and did not have biological children (Table 2). Men in the sample were somewhat older than their partners, but like the women, most were white, non-Hispanic, employed full-time, with no biological children. Men had somewhat less education and higher personal annual incomes than their female partners. Both the women and men in the sample had health-related quality of life scores that were at or better than the US average, as measured by the PROMIS system (4-item short forms for each domain), on which a score of 50 corresponds to the average for US adults with standard deviation (SD) of 10, and higher scores correspond to more of that domain [25, 26].

**Table 2:** Sample characteristics

	Women	Men	
Demographics, n (%)	*n* = 59	*n* = 59	*p*-value^a^
Age^b^			**<0.001**
< 30 years old	18 (30.5%)	11 (18.6%)	
30–34 years old	17 (28.8%)	16 (27.1%)	
35–37 years old	14 (23.7%)	13 (22.0%)	
≥ 38 years old	10 (16.9%)	19 (32.2%)	
Race/Ethnicity^c,d^			0.809
Asian, non-Hispanic	2 (3.4%)	1 (1.8%)	
Black/African American, non-Hispanic	1 (1.7%)	0 (0.0%)	
White, non-Hispanic	52 (89.7%)	51 (89.5%)	
Hispanic/Latino	3 (5.2%)	5 (8.8%)	
Religious Affiliation^d^			0.825
Protestant	26 (44.1%)	25 (42.4%)	
Catholic	23 (39.0%)	21 (35.6%)	
Other religion	3 (5.1%)	3 (5.1%)	
No religion	7 (11.9%)	10 (16.9%)	
Educational Attainment^b,c^			**0.021**
< College degree	13 (22.4%)	19 (33.3%)	
College degree (BA/BS)	22 (37.9%)	25 (43.9%)	
Advanced degree (MA, PhD, MD)	23 (39.7%)	13 (22.8%)	
Personal Income, in US dollars^b,e^			**0.047**
< $40,000	24 (41.4%)	10 (17.9%)	
$40,000 to $59,999	13 (22.4%)	19 (33.9%)	
$60,000 to $79,999	11 (19.0%)	13 (23.2%)	
≥ $80,000	10 (17.2%)	14 (25.0%)	
Employment Status^c,f^			0.804
Full-time employed	48 (82.8%)	49 (86.0%)	
Part-time employed, homemaker, other	10 (17.2%)	8 (14.0%)	
Have Biological Child(ren)^f,g^			0.754
Yes	7 (12.3%)	9 (16.1%)	
No	50 (87.7%)	47 (83.9%)	
PROMIS 4-item Short Forms, mean (SD)^h^			
Physical function	57.2 (5.0)	56.9 (6.0)	0.711
Anxiety	51.0 (8.0)	48.7 (8.0)	0.120
Depression	46.5 (6.7)	44.5 (6.5)	0.106
Fatigue	46.0 (7.9)	46.6 (8.6)	0.720
Sleep disturbance	47.6 (7.1)	46.7 (7.3)	0.564
Satisfaction with participation in social roles	55.1 (8.0)	53.4 (7.0)	0.237
Pain interference	45.2 (6.6)	46.4 (6.8)	0.377

^a^Bolded *p*-values indicate that male and female partners differed significantly within couples with α set to 0.05

^b^ *P*-value calculated using Wilcoxon’s signed-rank test, adjusted for ties

^c^Missing data from one woman and two men

^d^ *P*-value calculated using extended McNemar’s test

^e^Missing data from one woman and three men

^f^ *P*-value calculated using McNemar’s exact test

^g^Missing data from two women and three men

^h^ *P*-value calculated using paired *t*-test

### High and low family-building priorities

**Fig. 2:**
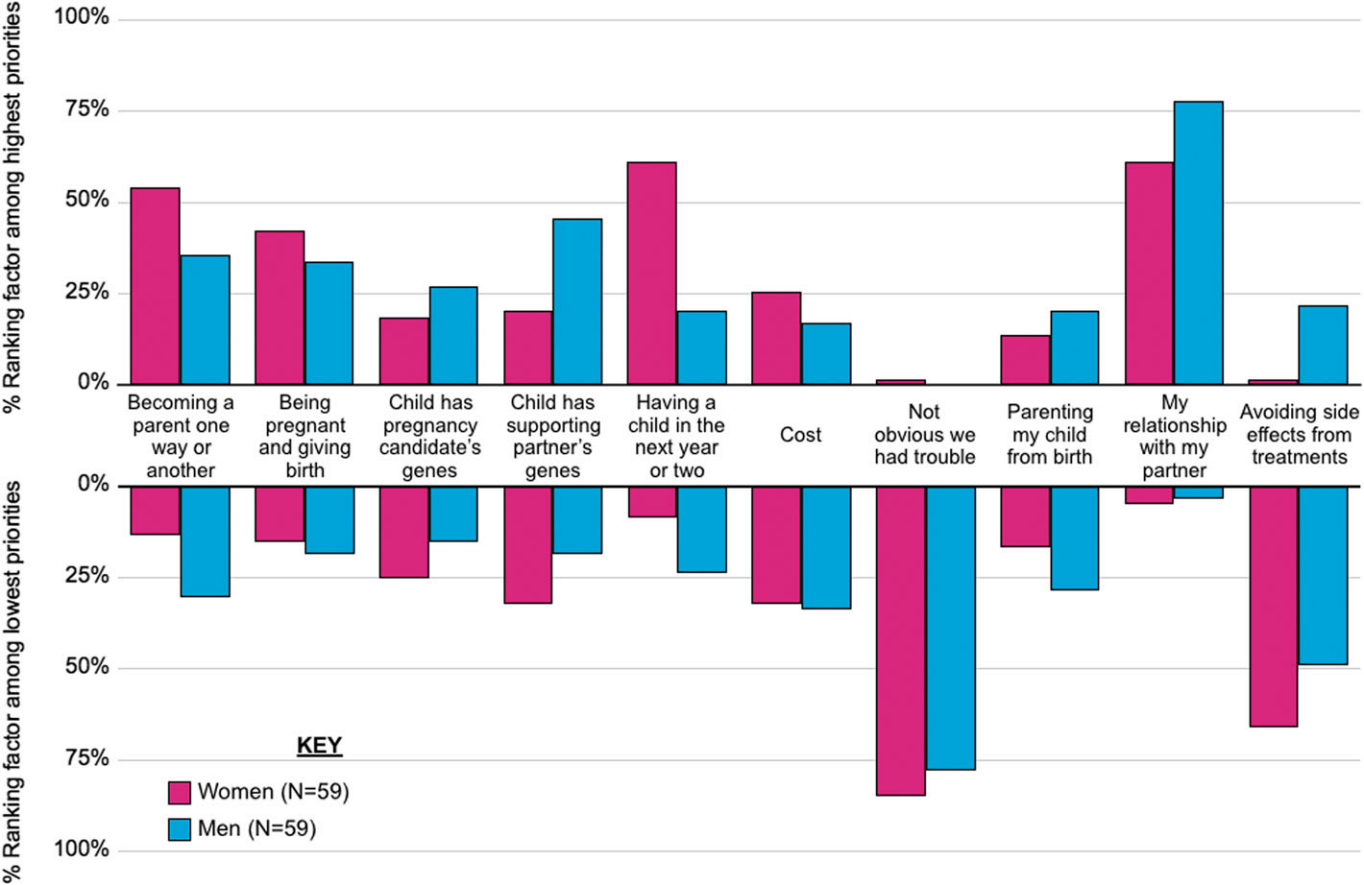
Highest and lowest priorities for family-building decision making by role. Displays percentages of women (*n* = 59) and men (*n* = 59) ranking each factor among their top 3 priorities (top half of figure) and bottom 3 priorities (bottom half of figure)

Figure 2 provides a snapshot of the importance of each of the factors by displaying the percentages of men and women who ranked each factor as a high priority (top half of figure) or a low priority (bottom half of figure). A majority of women ranked having a child in the next year or two, becoming a parent one way or another, and maintaining a close relationship with one’s partner as high priorities and building a family in a way that doesn’t make infertility obvious to others and avoiding side effects from treatment as low priorities. A majority of men ranked maintaining a close relationship with one’s partner as a high priority and building a family in a way that doesn’t make infertility obvious to others as a low priority. The importance of other factors proved to be more polarizing within each role, that is, at least one quarter of the group ranked the factor as a high priority while at least as many ranked the factor as a low priority. For women, cost was the single polarizing factor, and for men, the polarizing factor was becoming a parent one way or another.

### Alignment of family-building priorities within couples

Table 3 shows the alignment of priorities within couples. The results are similar to those observed in Fig. 2. Partners in nearly 1/3 of couples identically ranked the importance of building a family in a way that doesn’t make infertility obvious to others; in 27% of couples, both partners ranked this as their least important factor. Twenty-nine percent of couples were aligned about the importance of maintaining a close relationship with one another; in 25% of couples, both partners ranked this as the most important of the 10 factors. Two additional factors had alignment for nearly 1/5 of couples, namely that the woman gets to be the person who is pregnant and gives birth to a child and that the child has [man’s] genes.

**Table 3:** Priority alignment and differences within couples: *N* = 59 couples

		Differences^a^		
	Identical rankings	Woman ranked factor higher than man	Man ranked factor higher than woman	*p*-value^b^
Become a parent one way or another	6 (10.2%)	31 (52.5%)	22 (37.3%)	**0.015**
Carry the pregnancy and give birth	11 (18.6%)	27 (45.8%)	21 (35.6%)	0.312
Child has [woman’s] genes	6 (10.2%)	20 (33.9%)	33 (55.9%)	**0.025**
Child has [man’s] genes	11 (18.6%)	13 (22.0%)	35 (59.3%)	**<0.001**
Have a child in the next year or two	2 (3.4%)	42 (71.2%)	15 (25.4%)	**<0.001**
Cost	4 (6.8%)	31 (52.5%)	24 (40.7%)	0.322
Not obvious to others we had trouble	19 (32.2%)	14 (23.7%)	26 (44.1%)	0.111
Parent my child from birth	5 (8.5%)	29 (49.2%)	25 (42.4%)	0.283
Maintain close relationship with partner	17 (28.8%)	14 (23.7%)	28 (47.5%)	**0.034**
Avoid side effects from treatments	7 (11.9%)	14 (23.7%)	38 (64.4%)	**<0.001**

^a^ *P*-value calculated using Wilcoxon’s signed-rank test, adjusted for ties^b^ Bolded *p*-values indicate that male and female partners differed significantly within couples with α set to 0.05

### Differences in family-building priorities within couples

Within couples, men and women differed significantly in their prioritization of six of the factors: (1) becoming a parent one way or another was a higher priority for women compared to their partners (*p* = 0.015); (2–3) compared to their partners, men more highly prioritized genetic parentage, both passing on their own genes (*p* < 0.001) and passing on their partner’s genes (*p* = 0.025); (4) having a child in the next year or two was a higher priority for women than it was for their partners (*p* < 0.001); (5) while both men and women tended to highly prioritize maintaining a close relationship with their partner, within more couples men ranked this factor higher than women did (*p* = 0.034); and (6) compared to their partners, men more highly prioritized avoiding side effects from treatment (*p* < 0.001).

## Discussion

In the United States, securing a consultation with an RS requires some commitment and perseverance. Referral to an RS generally occurs after 12 months of unsuccessfully trying to conceive (or six months when a woman is 35 years of age or older) [27]; then couples often find that they must wait weeks or months for an opening in a specialist’s schedule. Those who persist often incur high out-of-pocket costs for the consultation [28], especially for the more than half of US adults who live in states (including Wisconsin, where this study was conducted) without an insurance mandate requiring any coverage for infertility diagnosis or treatment. Given all of this, it seems reasonable for an RS to presume that the patients and partners who make it to their clinic have made family building a high priority and therefore will prefer whatever course of action is most likely to lead to having a child. The findings reported in our study cast doubt on this presumption.

In this study, using a tool to assess family-building priorities in the context of infertility, we found that in the relatively early stages of exploring options to address infertility, that is, after scheduling but before attending an initial consultation with an RS, not all respondents ranked achieving parenthood one way or another among their highest priorities, and women tended to prioritize this factor more than men did. Furthermore, partners often held different ideas about the preferred timing of adding a child to their family, with women more often prioritizing having a child within the next year or two. We anticipated that cost might emerge as a key priority for patients and their partners because infertility treatments can be expensive and because, as noted above, Wisconsin does not mandate that health insurers cover medical care for infertility. Yet cost did not emerge as a top priority for most participants. However, a very clear message from the data is the emphasis placed on relationships: the majority of women and men prioritized the quality of their relationship, within couples more than a quarter of partners ranked it identically, and very few ranked their relationship among their lowest priorities, consistent with previous qualitative work [19].

If patient-centered care is a goal, RSs should be aware that a patient’s presence in their clinic does not necessarily imply that that patient (and/or their partner) is singularly focused on achieving parenthood. The results of a discrete choice experiment in Dutch and Belgian fertility clinics suggested that patients were willing to trade-off a higher pregnancy rate for more patient-centered care from physicians [29]. Scheduling and attending a consultation with an RS is just one of many family-building decisions patients and their partners will make if they proceed through fertility treatments, and in those subsequent decisions patients and their partners must balance their parenthood goals with other simultaneous and sometimes competing priorities, as also demonstrated in previous qualitative work [18]. The RS’s treatment recommendations must take into account patient and partner values and priorities along with their health history and test results. Our results highlight the need for RSs to be aware of the potential disconnect between patients and their partners on the importance of achieving parenthood and of the mutual importance placed on maintaining a close and satisfying relationship. Recognizing that the family-building priorities of patients and their partners commonly differ, RSs should encourage involvement of both partners in any treatment-related decisions. A concrete way to do this may be to recommend that both partners together attend not just the consultation, but also any follow-up appointments to review results and create treatment plans. As couples seek advice on treatment plans, it may be appropriate for the RS to raise directly the possibility of discrepant priorities and to explore with the couple how each partner’s priorities will or will not be served by the alternatives available. The RS might also recommend resources, such as counseling, when the two members of a couple struggle to reconcile disparate priorities.

This research was conducted at a single, suburban academic medical center in a convenience sample of new patients. It is possible that the priorities of participants and non-participants may differ, and the use of a convenience sample renders findings potentially subject to selection bias. While we instructed participants to complete the questionnaire separately from their partner, we cannot be certain that some did not discuss the questionnaire with their partner while completing it. In addition, the sample size is relatively small, limiting our ability to differentiate priorities by potentially relevant demographic and medical characteristics, such as infertility diagnosis or household income. Additional research is needed to place these findings in the context of other means of assessing the role played by cost in patients’ infertility treatment decisions. Better understanding of the complex associations among financial resources, infertility treatment, and ultimately outcomes, will illuminate the paths most likely to increase access to, and satisfaction with, care for all patients. Finally, future research should investigate the association between family-building priorities and various outcomes, including likelihood of achieving parenthood and long-term decisional satisfaction and regret.

## Conclusions

Understanding the extent to which both members of a couple typically do or do not share common priorities has important implications for providers who support patients in assessing the pros and cons of available family-building paths. RSs may consider utilizing the Family-Building Priorities Tool in the clinic to engage patients and their partners in a discussion about trade-offs and how different family-building paths align with patients’ and couples’ priorities. One fundamental consideration is that while medical procedures, including those for infertility, may involve just one patient, family building is typically a partnered activity, and the discussions and decisions that shape it should involve both prospective parents.

## Abbreviations

ART: Assisted reproductive technology
RS: Reproductive specialist
US: United States
